# Reference Beam Pattern Design for Frequency Invariant Beamforming Based on Fast Fourier Transform

**DOI:** 10.3390/s16101554

**Published:** 2016-09-22

**Authors:** Wang Zhang, Tao Su

**Affiliations:** National Laboratory of Radar Signal Processing, Xi’dian University, Xi’an 710071, China; sutao@xidian.edu.cn

**Keywords:** frequency invariant beamforming, reference beam pattern, fast Fourier transform, frequency invariant property

## Abstract

In the field of fast Fourier transform (FFT)-based frequency invariant beamforming (FIB), there is still an unsolved problem. That is the selection of the reference beam to make the designed wideband pattern frequency invariant (FI) over a given frequency range. This problem is studied in this paper. The research shows that for a given array, the selection of the reference beam pattern is determined by the number of sensors and the ratio of the highest frequency to the lowest frequency of the signal (RHL). The length of the weight vector corresponding to a given reference beam pattern depends on the reference frequency. In addition, the upper bound of the weight length to ensure the FI property over the whole frequency band of interest is also given. When the constraints are added to the reference beam, it does not affect the FI property of the designed wideband beam as long as the symmetry of the reference beam is ensured. Based on this conclusion, a scheme for reference beam design is proposed.

## 1. Introduction

**Figure 1:**
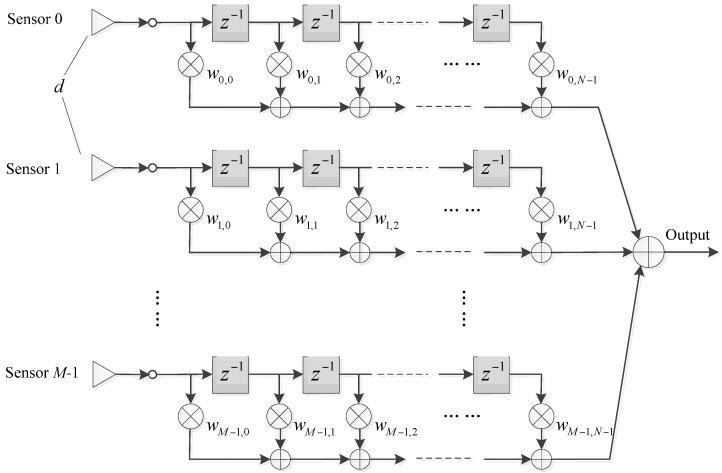
Structure of the wideband beamformer.

Wideband beamformers are extensively used in radar, sonar, speech processing and wireless communication. They usually comprise a sensor array with an FIR filter following each sensor, as shown in Figure 1. In the beamformer, the signals at each tap of the filters are multiplied by a weight coefficient, and then summed together to form the output.

A main drawback of the conventional wideband beamformer [1] is that the spatial resolution is proportional to the signal frequency. When the direction of the arrival (DOA) of the impinging signal deviates from the look-direction of the array, the signal suffers distortion (mainly amplitude distortion). This distortion is not desirable in some applications (such as microphone arrays [2]), and therefore the frequency invariant beamforming (FIB) technique was developed.

There are many FIB methods, such as the harmonic nesting method [3], least square method [4,5] and resampling method [6,7,8], but they are not flexible enough for controlling beam parameters or must be implemented in the frequency-domain. The introduction of the convex optimization method [9] solves nearly all these problems. In this method, the weight is directly optimized in the time-domain. In this process, the problem is expressed as a convex target function under convex various constraints on the beam [10,11], and then solved with the help of the convex optimization toolbox. Unfortunately, the existing convex optimization toolboxes cannot work well when the dimensions of the variable to be solved increase to a certain level, so it is not appropriate to use the convex optimization method for pattern synthesis when the number of sensors and FIR length are relatively large.

To overcome this problem, a class of methods, which is called FFT-FIB in this paper, is developed. This method was first proposed by Sekiguchi in [12], and then applied to the rectangular narrowband array for wideband beamforming by Ghavami [13]. Shortly after, Liu generalized this method and applied it to the general taps-delay-line (TDL) array and the sensor-delay-line (SDL) array with regular structure [14,15,16,17]. It exploits the Fourier transform relationship between the array pattern and the weight, and the weight can be obtained from the inverse discrete Fourier Transform. Because the inverse discrete Fourier transform can be realized by inverse fast Fourier transform (IFFT), it has considerable computational advantages over the convex optimization method.

In FFT-FIB, people must select a reference beam first and map it in the multiple-dimensional frequency-domain to construct the array spatio-temporal spectrum. Then, a multiple-dimension IFFT is applied to this spatio-temporal spectrum to obtain the filter coefficients, so how to select a reference beam for a wideband array working within a certain frequency range is the key to this algorithm. For example, if the selected reference beam is improper, the FI property of the designed wideband beam pattern will be not satisfactory. However, this problem is still unsolved till now.

The only work referring to this problem, to the best of our knowledge, is [12]. In this paper, the authors gave a vague conclusion “From many design examples, the value of *M* (sensor number) and *N* (filter length) need to be more than three times of *K* (length of the weight vector corresponding to the reference beam)”. It was then followed in nearly all other references on this method. However, this is only an empirical result for the experiment conditions given in that paper and cannot be simply extended to other conditions. So far, the existing literatures haven’t still given a universal conclusion for this problem.

In this paper, we study this problem and provide the reader a more universal conclusion. Based on a uniform linear array (ULA), we analyze the relationship between the FI property of the wideband beam, the number of sensors, and the length of the weight vector corresponding to the reference frequency. Our research shows that for the simple beam, the reference beam is determined only by the number of sensors and the ratio of the highest frequency to the lowest frequency of interest. And for a given reference beam, its weight length is determined by the reference frequency. When we add constraints to the reference beam, the FI property of the designed wideband beam is not affected as long as the constraints ensure the symmetry of the beam.

It’s worth noting that the FIR length and the number of the sampling grids in the frequency- domain also affect the FI property of the designed wideband beam, but this effect will be negligible when they increase to a certain level. Considering that our focus is the relationship between the reference beam pattern design and the FI property of the wideband beam, these two parameters are always set large enough in this paper. As a result, their effect will not be considered. In addition, this paper only deals with the broadside-mainbeam reference beam. The non-broadside case is not referred.

The paper is organized as follows: Section 2 simply introduces the FFT-FIB algorithm and gives some definitions to be used in the following sections. Section 3 shows the inadequacy of the existing conclusion. In Section 4, the relationship between the FI property of the wideband beam pattern, the number of sensors and the length of the weight corresponding to the reference beam is analyzed, for the simple reference beam. Based on these conclusions, we study the reference beam design with constraints in Section 5. Some experiments are provided in Section 6 to verify our research. And conclusions of this paper are drawn in Section 7.

## 2. Definitions

Consider a wideband ULA with *M* sensors and FIR filter length *N* as shown in Figure 1. Assume all the sensors are isotropic, and the frequency range of interest is $f\in \left[f_{L},f_{H}\right]$, where *f*_L_ and *f*_H_ are the lowest and highest frequency of interest. The inter-element spacing is *d* = *c*/2*f*_H_, where *c* is the propagation velocity of the wave. To avoid the spectrum aliasing problem and without loss of generality, we set *f_s_* = 2*f*_H_.

The beam pattern of the array is given by:

(1) $$p(\Omega_{1},\Omega_{2})=\sum_{m=0}^{M-1}e^{-jm\Omega_{1}}\sum_{n=0}^{N-1}w_{m,n}e^{-jn\Omega_{2}}$$

where Ω_1_ = (2*πfd*sin*θ*)/*c*, Ω_2_ = 2π*f/f_s_*, *θ* is the angle from the broadside, and *w_m,n_* is the coefficient of the *n*th tap in the filter following the *m*th sensor. Then the FFT-FIB algorithm can be summarized as follows [14]:

(1) Discretize Ω_1_, Ω_2_ into *X*_1_ and *X*_2_ points, respectively, where Ω_1_, Ω_2_ ∈ [−*π,π*), *X*_1_ ≥ *M* and *X*_2_ ≥ *N*. For a given reference beam:
  (2) $$F(\sin\theta )=\sum_{k=-(K-1)/2}^{(K-1)/2}w_{k}e^{\frac{-j2\pi f_{\mathrm{ref}}kd\sin\theta }{c}}$$
  where *f*_ref_ is the reference frequency, *w_k_* is the weight function corresponding to the reference beam and will be called reference weight in the following paper, and *K* is the length of *w_k_*, we have:
  (3) $$p(\Omega_{1},\Omega_{2})=\left\{ \begin{array}{l} \begin{array}{cc} F(\Omega_{1}/\Omega_{2}), & \left|\Omega_{1}/\Omega_{2}\right|\leq 1 \end{array}\\ \begin{array}{c} \begin{array}{cc} \;0\;, & \;\mathrm{otherwise} \end{array} \end{array} \end{array}\right.$$

**Figure 2:**
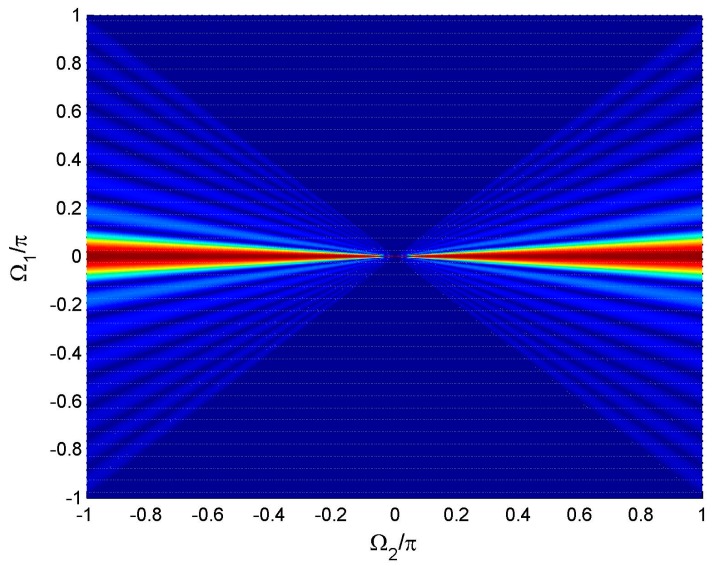
Spatio-temporal spectrum of the signal.

The two-dimensional (2-D) figure of *p*(Ω_1_,Ω_2_) in the Ω_1_ − Ω_2_ plane is shown in Figure 2. It can be seen that the spatio-temporal spectrum of the signal lies between the lines Ω_1_ = Ω_2_ and Ω_1_ = −Ω_2_.

(2) Apply 2-D IFFT to *p*(Ω_1_,Ω_2_) so as to obtain the 2-D weight function *w_m,n_* where |*m*| ≤ (*X*_1_−1)/2 and |*n*| ≤ (*X*_2_−1)/2, then truncate *w_m,n_* to the prescribed length along the *m* axis and *n* axis, respectively.

Here we define the ratio of the highest frequency to the lowest frequency (RHL):

(4) $$\mathrm{RHL}=f_{H}/f_{L}$$

It represents the required frequency range over which the designed wideband beam is FI. To investigate the FI property of the wideband beam in the direction *θ*, we then define two performance functions. One is the relative spatial magnitude response variation (RSRV) function:

(5) $$\begin{array}{l} \mathrm{RSRV}(\theta )=\frac{1}{J}\sum_{j=1}^{J}\frac{\left|\left|p(\theta ,f_{j})\right|-\left|F(\sin\theta )\right|\right|}{\left|F(\sin\theta )\right|}\\ \begin{array}{cc} \begin{array}{c} \;\theta \in [-90{}^{\circ},90{}^{\circ}], \end{array} & \;f_{j}\in [f_{L},f_{H}] \end{array} \end{array}$$

where *J* is the number of the uniformly discretized grids over [*f*_L_,*f*_H_]. It reflects the mean fluctuation of the designed wideband beam pattern relative to the reference beam in the direction *θ* over [*f*_L_,*f*_H_].

The other is the total spatial magnitude response variation (TSRV):

(6) $$\begin{array}{l} \mathrm{TSRV}=\frac{1}{LJ}\sum_{l=1}^{L}\sum_{j=1}^{J}\left|\left|p(\theta_{l},f_{j})\right|-\left|F(\sin\theta_{l})\right|\right|\\ \begin{array}{cc} \;\theta_{l}\in [-90{}^{\circ},90{}^{\circ}], & \;f_{j}\in [f_{L},f_{H}] \end{array} \end{array}$$

where *L* is the number of the uniformly discretized grids over [−90°,90°]. It reflects the total fluctuation between the designed wideband beam and the reference beam over [*f*_L_,*f*_H_].

## 3. Problem Formulation

As for the selection of *K*, a common practice for the authors in [12,17] is setting *M* ≥ 3*K* in their experiments and by which a satisfying result is indeed obtained. However, there is hardly any reason given for this practice. In [12], the authors mentioned that it is only an empirical result. In this section, we shall, by two examples, show that this practice is not accurate enough for other conditions.

### 3.1. First Example

The aim of our first example is to show that the condition *M* ≥ 3*K* is not precise enough for different RHL. Consider a uniform-weighting beam which is extensively used for the reference beam in [12,13,14,15,16,17]:

(7) $$F_{1}(\sin\theta )=\sum_{k=-15/2}^{15/2}e^{-j\pi k\sin\theta }$$

**Figure 3:**
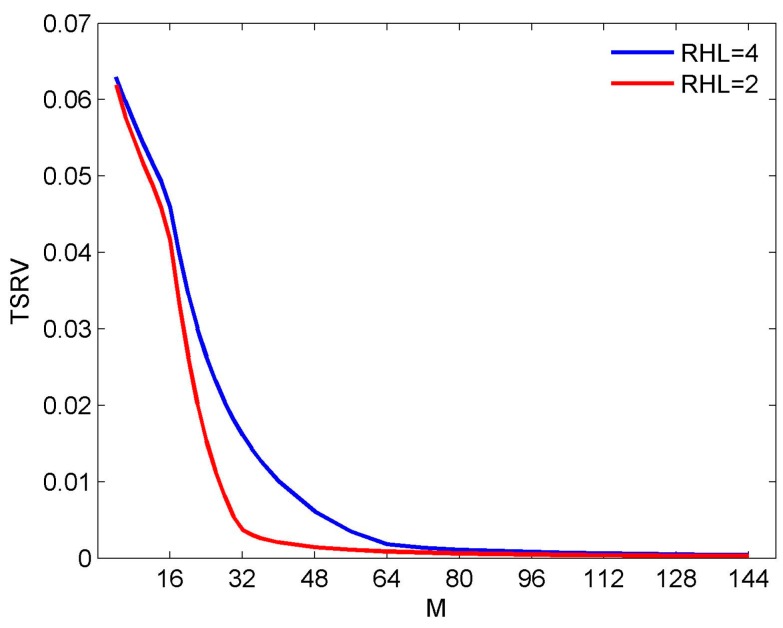
TSRV versus the number of sensors for different RHL.

Compared with Equation (2), it is seen that *f*_ref_ = *f*_H_ and *K* = 16 in Equation (7). Figure 3 shows the curve of the TSRV for RHL = 4 and RHL = 2, respectively. Generally speaking, the RSRV would be more meaningful as it shows the relative fluctuation of the beam in each direction. The reason we choose TSRV here is that it’s not practical to plot the RSRV for each trail. Although the value of the TRSV cannot give us the detailed information, it can also reflect the trend of the FI property versus RHL. In Figure 3, the curve converges at about *M* = 64 and *M* = 40, which are 4*K* and 5*K*/2, respectively. Obviously, the condition *M* ≥ 3*K* is not accurate enough to explain this result.

### 3.2. Second Example

The aim of the second example is to show that the condition *M* ≥ 3*K* is also not precise enough for different reference frequencies, even if the reference beam and RHL are the same. Commonly, we don’t focus on the exact shape of the desired beam pattern, but rather we are interested in some other quantities, such as mainbeam width (MW), or sidelobe level (SLL). For different reference frequencies, there usually exists different weights corresponding to a beam with the same technique index, and the length of them can be either equal or unequal.

To make the reader clear, we then give two other uniform-weighting beams besides *F*_1_(sin*θ*):

(8) $$F_{2}(\sin\theta )=\sum_{k=-31/2}^{31/2}e^{-j\frac{2\pi k\sin\theta }{3}}$$

It can be seen that *f*_ref_ = *f*_H_/2 and *K* = 32 in Equation (8).

(9) $$F_{3}(\sin\theta )=\sum_{k=-47/2}^{47/2}e^{-j\frac{\pi k\sin\theta }{3}}$$

**Figure 4:**
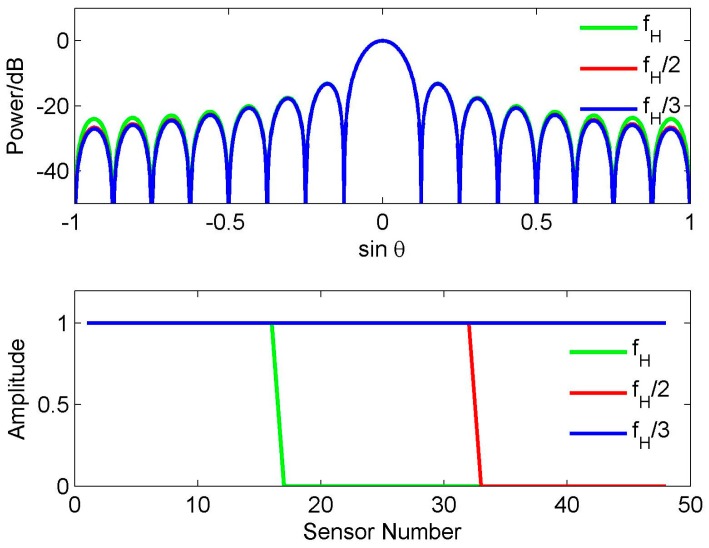
Beam patterns and weight function for different reference frequencies.

**Figure 5:**
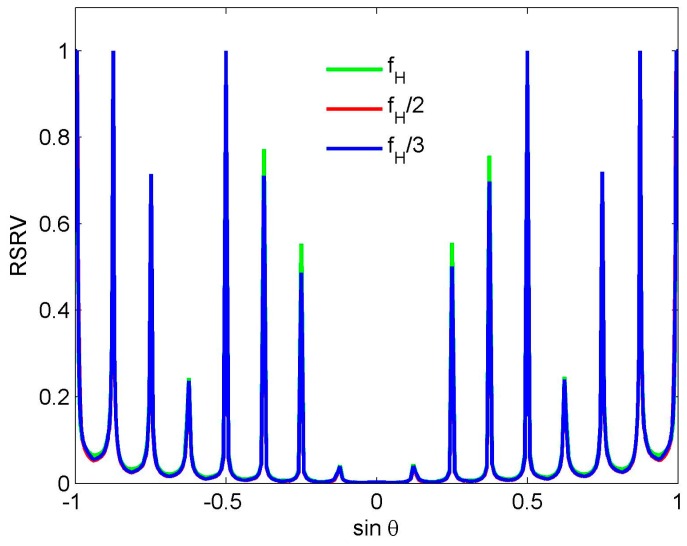
RSRV of the wideband beams designed by using the reference beams in Figure 4.

It can be seen that *f*_ref_ = *f*_H_/3 and *K* = 48 in Equation (9). Figure 4 shows the beam (the top half) and the corresponding weights (the bottom half) in Equations (7)–(9). To distinguish three different weights, the weight vector at *K* = 16,24 are padded zeros to make their length equal to 48. It is observed that although these weights have different length, the beam pattern corresponding to them has the same beamwidth and peak sidelobe level. Note that although there is a little difference near the end-fire area, these three beams are usually seen as a same beam in the real application. Using these reference beams, we obtain three wideband beams patterns, respectively. Their RSRV curves are shown in Figure 5. Due to the RSRV value near the nulls is usually larger than that in other areas, for convenience, the value larger than 1 is set to 1. It can be seen that the variation of the weight length does not nearly affect the FI property. If the number of sensors is 48, we therefore have *M* = 3*K*, 2*K* and *K*, respectively. It is clear that *M* ≥ 3*K* is also not precise enough to explain this phenomenon.

## 4. Reference Beam Pattern Design for Simple Beams

In the proceeding section, we simply show that the condition *M* ≥ 3*K* is not precise enough for the reference beam design in FFT-FIB. In this section, we propose a more common guidance for simple reference beams. The simple beam mentioned here contains the uniform-weighting beam, Chebyshev beam, Taylor beam, and other beams generated by the window functions. A common ground of them is that their shape is regular and symmetric. Before starting our work, we give some preliminary analysis.

Denote:

(10) $$F_{m}(\Omega_{2})=\sum_{n=0}^{N-1}w_{m,n}e^{-jn\Omega_{2}}$$

Then Equation (1) can be rewritten as:

(11) $$p(\theta ,\Omega_{2})=\sum_{m=0}^{M-1}e^{\frac{-jm\Omega_{2}f_{s}d\sin\theta }{c}}F_{m}(\Omega_{2})$$

It is seen from Equation (11) that the weight vector of the beam pattern corresponding to each frequency bin is composed of the value of the frequency response function of *M* FIR filters at this frequency bin. That is, if we apply an IFFT to *p*(Ω_1_,Ω_2_) in Equation (3) along the Ω_1_ axis, the result:

(12) $$\begin{array}{cc} P(m,\Omega_{2})={\mathrm{IFFT}}_{\Omega_{1}}[p(\Omega_{1},\Omega_{2})], & m=-\left(X_{1}-1\right)/2,\cdots ,\left(X_{1}-1\right)/2 \end{array}$$

is the weight vector of the designed wideband beam, before truncation, at the frequency Ω_2_.

For the convenience of our analysis, we further divide the FFI-FIB algorithm into five steps in detail, as follows:

- Pattern mapping.
- IFFT in the Ω_1_ dimension.
- IFFT in the Ω_2_ dimension.
- Truncation in the *m* dimension.
- Truncation in the *n* dimension.

According to the property of two-dimensional FFT, the finally result is not affected when the order above is changed as A-B-D-C-E. It means that in Equation (12) only the data within *m*∈[−(*M*−1)/2,(*M*−1)/2] is actually useful for the next steps. As a result, we denote:

(13) $$\begin{array}{c} w(k)=P(m,\Omega_{2k}),\;m=-\left(M-1\right)/2,\cdots \left(M-1\right)/2,\;\Omega_{2k}\in [-\pi ,\pi ) \end{array}$$

where Ω_2*k*_ is the *k*th sampling frequency bin in the Ω_2_ dimension. Then based on Equations (10)–(13), it is seen that to analyze the FI property of the designed wideband beam pattern, we need only to discuss whether different *w*(*k*) satisfies the FI condition, or more generally, which condition should be satisfied for an *M*-sensor narrowband array to be FI at different frequencies, so in the following part, we shall, based on a narrowband array, analyze the relationship between the FI property of the designed wideband beam pattern, the number of sensors, and the length of the reference weight vector.

### 4.1. Relationship between K and f_ref_

For convenience, we denote *F*(sin*θ*) as *F*(sin*θ*,*f*_ref_) to highlight the effect of the reference frequency. Assume two different reference frequencies *f*_ref1_ and *f*_ref2_, the corresponding weight *w*_1_(*k*) and *w*_2_(*k*), and the weight vector length *K*_ref1_, *K*_ref2_. If *F*(sin*θ*,*f*_ref_) is independent of frequency (frequency invariant), the following condition:

(14) $$w_{1}(k)=\alpha w_{2}(\alpha k)$$

where *α* = *f*_ref1_/*f*_ref2_, must approximately hold [8]. Equation (14) shows that to obtain a FI wideband beam, the effective relative aperture length corresponding to each frequency component must be equal, and the real aperture length at *f*_ref1_ is 1/*α* times of that at *f*_ref2_. It means that to make the beam FI, the weight vector at a higher reference frequency is always no longer than that at a lower reference frequency (i.e., if *α* ≥ 1, then *K*_ref1_ ≤ *K*_ref2_), which also interprets the result in Figure 4 and Figure 5.

**Figure 6:**
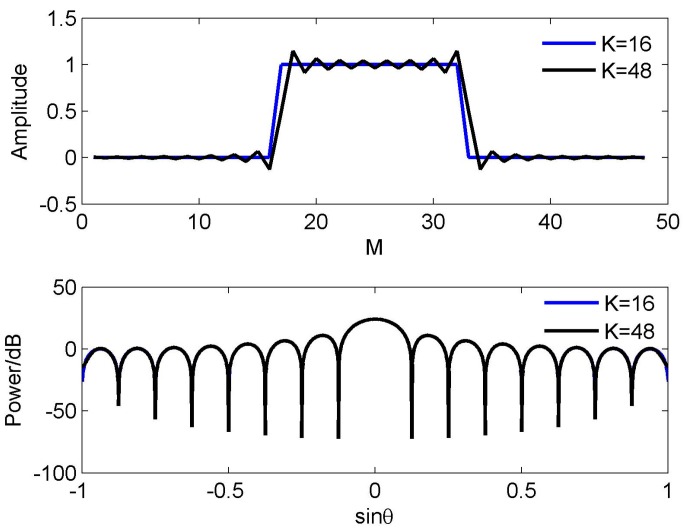
Different weight length for a same reference beam and a same reference frequency.

Thus, it can be deducted that *K* tends to become larger as *f*_ref_ decreases, but it is not a necessary result. Generally, even for a same reference beam and a same reference frequency, the length of the weight vector can also be different. Let us illustrate this point by an example. Assume *M* = 48, and *f*_ref_ = *f*_H_. Figure 6 shows two weight vectors **w**_1_ and **w**_2_ (the top half) and the corresponding beam (the bottom half). It is seen that the length of **w**_1_ and **w**_2_ are *K*_ref_ = 16,48, respectively (also, to distinguish these two vectors, **w**_1_ is padded with zeros to make its length reach 48). However, the corresponding beams can be seen as the same in the application. In fact, the effective aperture length of **w**_1_ and **w**_2_ are equal. The reason why the length of **w**_1_ is less than **w**_2_ is that there is no energy leakage in **w**_1_.

Since the reference frequency has little effect on the FI property for a given desired beam pattern, in the following analysis, we always assume *f*_ref_ = *f*_H_, and the reference weight vector has the same form as **w**_1_ (no energy leakage), which leads to the smallest *K*_ref_, and simply use *K* instead of *K*_ref_.

### 4.2. Relationship between FI Area and K

Firstly, it’s necessary to introduce the Nyquist sampling theorem on pattern sampling [18].

Denote ω = *f*_ref_*kd*/*c* *f*_H_, *u* = sin*θ*. Then Equation (2) is rewritten as:

(15) $$F(u)=\sum_{k=-(K-1)/2}^{(K-1)/2}w_{k}e^{-j2\pi \omega u}$$

Due to *f*_ref_ = *f*_H_, −*K*/4 ≤ *ω* ≤ *K*/4. According to the Nyquist sampling theorem, when *F*(*u*) is sampled, the sampling interval must satisfy:

(16) Δu≤2/K.

Because −1 ≤ *u* ≤ 1, the number of the samples needed to recover the beam pattern without distortion must be no less than *K*. Now we analyze the FI area of the designed wideband beam.

Assume *K* is given. It can be concluded from Equation (3) that as Ω_2_ decreases, the beam pattern is gradually compressed linearly along the Ω_1_ axis, and meanwhile the sample number in the visible area reduces linearly, which is shown in Figure 2. Combining the desired beam pattern mapping with the IFFT operation along the Ω_1_ axis, we find that it is in fact an “FFT interpolation [8]” process for the reference weight, which realizes Equation (14). According to the Nyquist sampling theorem and interpolation theorem, the FI property of the designed wideband beam at the frequency Ω_2_ is ensured, only if the samples of *p*(Ω_1_,Ω_2_) along the Ω_1_ axis are more than *K*. Thus, the frequency range corresponding to the FI area of the wideband beam before truncation is approximately:

(17) $$f_{b}=[f_{H}K/X_{1},f_{H}]$$

In FFT-FIB, the coefficients after IFFT will be truncated to *M*, and therefore the FI area after truncation is of more significance. From Equation (14), it can be deducted that the lowest frequency at which the relative aperture length is equal to that at *f*_H_ is *f*_H_*K*/*M*. As a result, for a given *M* and *K*, the frequency range corresponding to the FI area of the designed wideband beam after truncation is approximately:

(18) $$f_{a}=[f_{H}K/M,f_{H}]$$

### 4.3. Relationship between K and RHL

We know that the real aperture length of the weight vector, to meet Equation (14), becomes longer as the frequency decreases. Due to the length of the real array is finite, the FI property of the wideband beam at a certain frequency cannot be ensured if the real aperture length at this frequency is longer than the aperture length corresponding to the real array. For a given RHL, it therefore can be concluded from Equation (14) that to ensure the FI property over the whole frequency range of interest, the minimum length of the reference weight vector is approximately:

(19) K≤floor(M/RHL),

where “floor(.)” represents rounding the element towards zero.

### 4.4. Discussion

Equations (17)–(19) are the main conclusions we obtained for the reference beam design. However, it should be noted that when giving these equations, we always use the words “approximately”. The reasons are as below:

(1) Distortion in the reference pattern sampling. In the process of the reference pattern mapping in Equation (3), as Ω_2_ close to *f*_H_*K*/*X*_1_, there will be many zero or close-to-zero samples. In this case, the samples of the beam pattern are incapable of representing the beam pattern precisely, which makes the FI property of the wideband pattern before truncation deteriorate as Ω_2_ is close to *f*_H_*K*/*X*_1_ in Equation (17).
(2) Error resulting from the truncation. As mentioned before, the weight vectors at different frequencies are the stretched or compressed versions of the reference weight vector, and obtained by interpolation and truncation. Usually, for a set of weights generated by this way, the length of them before truncation are infinite (energy leakage exists), so the error after truncation always exists. Due to the fact the real aperture length of the weight vector at a lower frequency is larger than that at a higher one, the truncation error in it is also larger than that in the higher one, so it can be concluded that the FI property of the wideband beam pattern in the lower frequency range is usually poorer than that in the higher frequency range. Especially when *K* = floor(*M*/RHL), the effective aperture length of the weight vector at about *f*_L_ is nearly equal to the truncated array aperture length, which will lead to a considerable truncated error and degrade the FI property in this frequency range.

It can be also concluded from Equations (17)–(19) that, for a given *K*: (i) the FI area, after truncation, of the designed wideband beam is always no larger than that before truncation; (ii) increasing the number of the sampling grids along the Ω_1_ axis contributes to improving the FI property before truncation (it has the same effect as adding more sensors); (iii) the FI property after truncation is independent of the sampling grid number along the Ω_1_ axis. Increasing the sampling density along the Ω_1_ axis cannot improve the FI range after truncation obviously, except for increasing the computational burden. However, when RHL is close to 1, the number of the sampling grids along the Ω_1_ axis should better be a little larger than the number of sensors, so as to avoid the FI property degradation resulting from the sampling error of the reference beam.

In the last part of this section, based on Equations (17)–(19), some useful conclusions for the simple reference beam pattern design in FFT-FIB is given as below:

(1) For a given array with *M* sensors, the reference beam pattern is almost completely determined by *M* and RHL, as shown in Equation (19). According to Equation (19), it can be deducted that for a fixed *K*, increasing *M* means a larger FI area of the designed wideband beam pattern. Or, for a fixed RHL, increasing *M* means a potential larger *K*. Here we should state that in Section 5 of [12] the authors also gave a similar conclusion that “by increasing *M*, the range of the frequencies over which the beam patterns coincide becomes larger, especially at lower frequencies”. But our conclusion Equation (19) is more precise. It provides a theoretical and quantitative result. For example, for any given *M* and RHL, people can quickly determine the range of feasible *K* referring to Equation (19) without doing experiments. However, the conclusion in [12] is relative obscure and lack of theoretical support.
(2) For a given beam pattern, *K* only depends on *f*_ref_, and the minimum of *K* is always obtained at reference *f*_H_.
(3) The upper bound of the minimum of *K*, to make the wideband pattern frequency invariant over the whole frequency band, is floor(*M*/RHL). To ensure the FI property at the frequency close to *f*_L_, *K* should better be a little less than floor(*M*/RHL).
(4) For a given reference beam pattern:
  - If RHL is an integer, a wideband beam with a little lower designing computation complexity will be obtained when *f*_ref_ equals *f*_H_ and *K* equals floor(*M*/RHL).
  - If RHL is not an integer, a wideband beam with a narrower mainbeam is obtained when *f*_ref_ equals *f*_L_ and *K* equals *M*.

## 5. Reference Beam Pattern Design with Constraints

In Section 4, we give a common guidance for the reference beam design in FFT-FIB. However, the performance of these beams, in most cases, cannot satisfy the application requirement. Most commonly, we should prescribe some performance indexes for the reference beam, such as mainbeam width, sidelobe level, or null constraints, which make the reference beam with a much more complex shape. In this section, we make a further analysis, and then propose some practical suggestions for complex reference beam design.

### 5.1. Preliminary Analysis

It should be firstly pointed out that the conclusion for the simple beam in Section 4 is significant, because almost each complex beam can be seen as an approximation of a simple beam subject to some constraints in the following form:

(20) $$\begin{array}{l} \begin{array}{cc} \underset{w}{\min}. & \left\|w^{H}a_{\theta }-w_{0}^{H}a_{\theta }\right\| \end{array}\\ \begin{array}{cc} \begin{array}{cc} s.t. & Q_{i}(w \end{array})=f, & i=1,2,\cdots I \end{array}\\ \begin{array}{cc} \begin{array}{c} \;Z_{t}(w) \end{array}\leq g, & t=1,2,\cdots T \end{array} \end{array}$$

where **a**_θ_ is the steering vector, **w**_0_ is the weight vector corresponding to a simple reference beam (for example, if the nulls are of more interest to us, **w**_0_ could be a uniform weight vector, and if the SLL is of more interest, **w**_0_ could be a Chebyshev weight vector), **Q***_i_*(**w**) is the hard-constraint function matrix which could be either linear or nonlinear (such as, quadratic), **Z***_t_*(**w**) is the soft-constraint function matrix which can also be linear or non-linear, **f** and **g** are the equality constraint condition vector and non-equality constraint condition vector. From Equation (20), it is clear that the complex reference beam for the FFT-FIB must satisfy Equations (17)–(19) and other conclusions in Section 4 first.

Intuitively, the constraints could not have affected the FI property of the wideband beam pattern for FFT-FIB as long as the conclusions in Section 4 are satisfied. In Section 4, we said that the desired beam pattern mapping combining the IFFT operation along the Ω_1_ axis is in fact “a pattern sampling first and then interpolation process” for the reference weight. And according to the Nyquist sampling theorem, a little more than *K* samples is absolutely enough to recover the pattern, regardless of the shape of beam pattern. It means that there seems no reason for a reference beam, which satisfies Equations (18) and (19), to make the designed wideband beam with a poor FI property in FFT-FIB. However, it is not this case. This problem will be formulated in the following parts.

### 5.2. FIB Case

**Figure 7:**
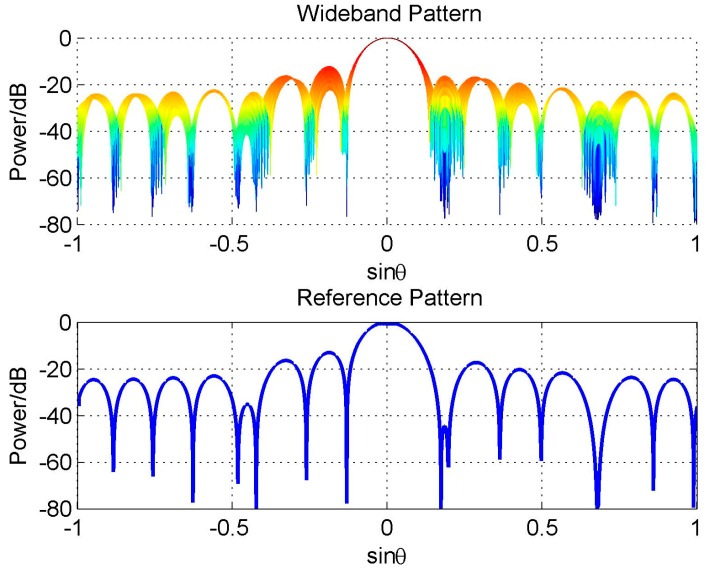
Beam pattern for asymmetric null constraints.

We firstly discuss a simple constraint form, the hard linear constraint, and give an example here. Here we also consider the uniform-weighting reference beam as Equation (7), but differently, we add three linear hard null constraints in the direction 42.6°, 10° and −25°. The algorithm in [19] is used to design the reference beam. Figure 7 shows the designed reference beam and the consequent wideband beam pattern designed by FFI-FIB. We only plot its two-dimensional (2-D) figure of the wideband beam, because it gives a more clear sight of its FI property which is not satisfying as we see.

**Figure 8:**
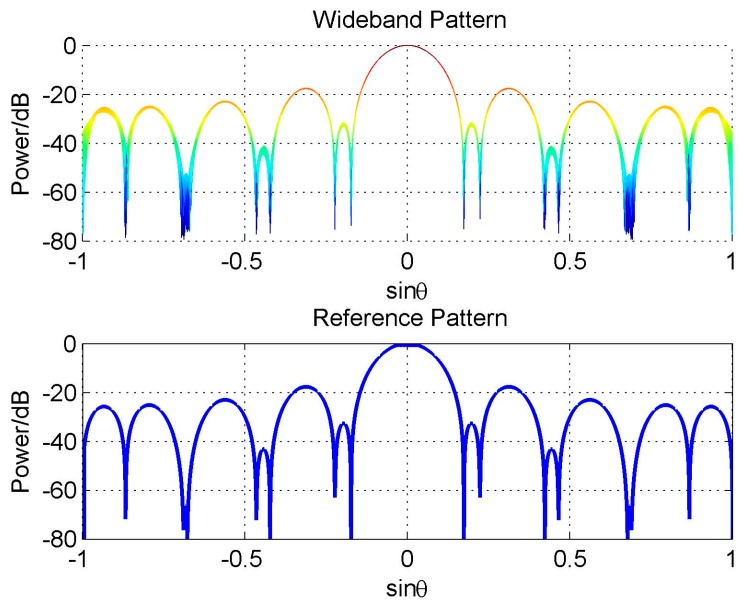
Beam pattern for symmetric null constraints.

Actually, the reason for this problem is not the constraint itself, but that adding constraints destroys the symmetry of the reference pattern (see Figure 7), which leads to the discontinuity close to the frequency *π* and degrades the FI property of the designed wideband beam [20]. If we add another three hard null constraints in the direction −42.6°, −10° and 25° to make the reference beam pattern symmetric, the FI property will be improved greatly, as shown in Figure 8.

Now based on the analysis above, we give our first conclusion for complex reference beam pattern design with linear hard constraints, as below:

If the linear hard constraints do not affect the symmetry of the reference beam, they must be symmetric about θ = 0°.

Now we discuss the relationship between *K* and the hard constraint number. Assume the hard linear constraint number is *C*. We know, a linear hard constraint will consume a degree of freedom completely, and at most *K* − 1 hard linear constraints can be added for an array with *K* sensors. In addition, to make the beam symmetric, two degrees of freedom must be consumed for a hard constraint. Combining with (19), we have the following relationship:

(21) 2C+1≤K≤M/RHL,

Unfortunately, in most cases, the constraint has a complex form, and it is difficult to estimate how many degrees of freedom the constraints consumes. For example, it is difficult to estimate the number of the degrees of freedom consumed by a soft quadratic constraint in beam design. In this case, Equation (21) is not practical. But based on the perspective of the symmetry of the reference beam, we can, at least, give a rough conclusion, as below:

If the reference beam is symmetric about θ = 0°, it is always suitable for FFT-FIB.

Now our task becomes designing a symmetric reference beam under various constraint conditions. To solve this problem, the filter design method can be applied. According to the filter design theory, if the weight vector is symmetric, the corresponding beam pattern must be symmetric. Then, a general scheme for the reference beam design for FFT-FIB is given, as below:

*Scheme for designing reference beam pattern with constraints*: Assume **W**_ref_ is the *K*-dimension reference weight vector, *w*_0_ is a constant, and **w**_h_ = [*w*_1_, *w*_2_,…, *w_H_*], where *H* = *K*/2, if *K* is even; or *H* = (*K* − 1)/2, if *K* is odd. The scheme for the reference beam design is summarized as follows:

(i) Calculate RHL and *K*, according to Equations (4) and (19), respectively.
(ii) Construct the following weight structure:
  (22) $$W_{\mathrm{ref}}=\left\{ \begin{array}{l} \begin{array}{cc} \;[\begin{array}{cc} w_{h}, & w_{-h} \end{array}], & \begin{array}{cc} \begin{array}{ccc} if & K & \mathrm{is} \end{array} & \mathrm{even} \end{array} \end{array}\\ {}[\begin{array}{cc} \begin{array}{cc} w_{h}, & w_{0}, \end{array} & w_{-h} \end{array}],\begin{array}{cc} \begin{array}{ccc} if & K & \mathrm{is} \end{array} & \mathrm{odd} \end{array} \end{array}\right.,$$
  where **w**_−h_ is the vector obtained by flipping **w**_h_ in the left-right direction.
(iii) Solve the problem:
  (23) $$\begin{array}{l} \begin{array}{cc} \underset{W_{ref}}{\min}. & A(W_{\mathrm{ref}}) \end{array}\\ \begin{array}{cc} \begin{array}{cc} s.t. & Q_{i}(W_{\mathrm{ref}} \end{array})=f, & i=1,2,\cdots I \end{array}\\ \begin{array}{cc} \begin{array}{c} \;Z_{t}(W_{\mathrm{ref}}) \end{array}\leq g, & t=1,2,\cdots T \end{array} \end{array},$$
  where *A*(**W**_ref_) is the target function. In Equation (23), all the solutions are suitable for the reference weight in FFT-FIB. If no solution exists for Equation (23), maybe the FFT-FIB is not suitable for our design target. Then we should add more sensors, or turn to other FIB methods.

### 5.3. Constant Beamwidth Case

In the proceeding part, we referred to the FI property in the whole spatial domain. However, the FI property in the sidelobe, in many cases, is not of much interest, and what we care about is only the FI property in the main-beam (the constant beamwidth case). In this case, the condition in Equation (23) is too strict. We need only ensure the symmetry around the main-beam, and meanwile suppress the other sidelobe below a certain level, because the effect of the discontinuity of the pattern on the FI property is not obvious in the area where the beam level is very low. That is to say, if the constraints occur in the area where the beam level is very low, and was unable to raise the beam level too much, it will not affect the FI property in the mainlobe obviously, even if it is not symmetric. Based on the analysis above, some conclusions on the complex reference beam pattern design for FFT-FIB are given.

In FFT-FIB, it’s better to make the reference beam symmetric so as to ensure a good FI property of the designed wideband beam. But it may also consume many degrees of freedom. The number of the degrees of freedom which could be used for constraint is at most *K*/2−1. For the constant beamwidth case, this condition could be much looser. To obtain a good FI property within the mainbeam, we need only ensure the symmetry around the mainbeam, and suppress the SLL as low as possible.

## 6. Experiments

Two groups of experiments are implemented to verify our results obtained in Section 4 and Section 5. The aim of the first group is to verify Equations (17)–(19) in Section 4.

**Figure 9:**
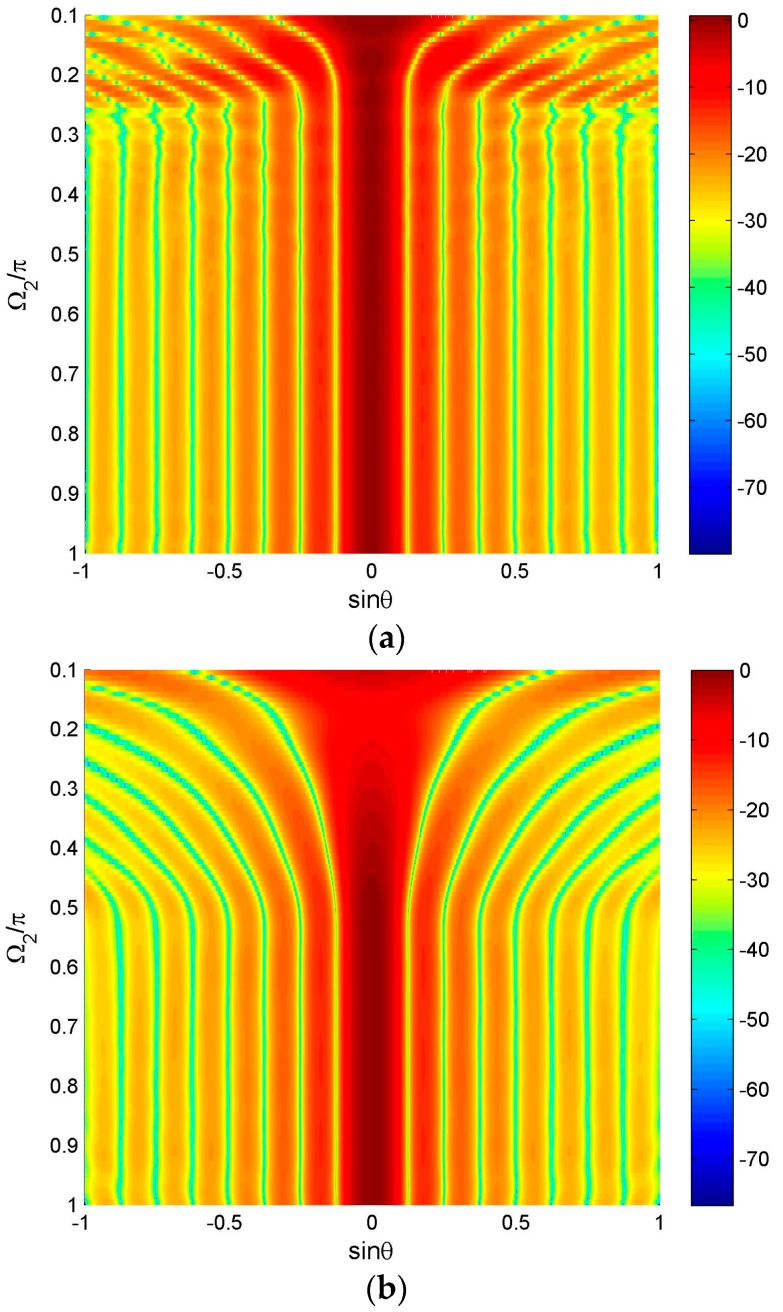
The wideband beam pattern designed by FFT-FIB. (**a**) Before truncation; (**b**) After truncation.

Assume *M* = *N* = 32, *X*_1_ = *X*_2_ = 64, RHL = 2, and *K* = 16. The reference beam is still the uniform-weighting beam. Figure 9 shows the spatial-frequency-plane view of the wideband pattern before truncation and after truncation, respectively. The frequency range of the FI area before truncation is about [0.25*π*,*π*], and the FI property degrades more or less in the frequency range about [0.25*π*,0.3*π*]. The FI area after truncation is about [0.5*π*,*π*] and the FI property begins to degrade in the frequency range about [0.5*π*,0.54*π*]. The result is consistent with Equations (17) and (18).

**Figure 10:**
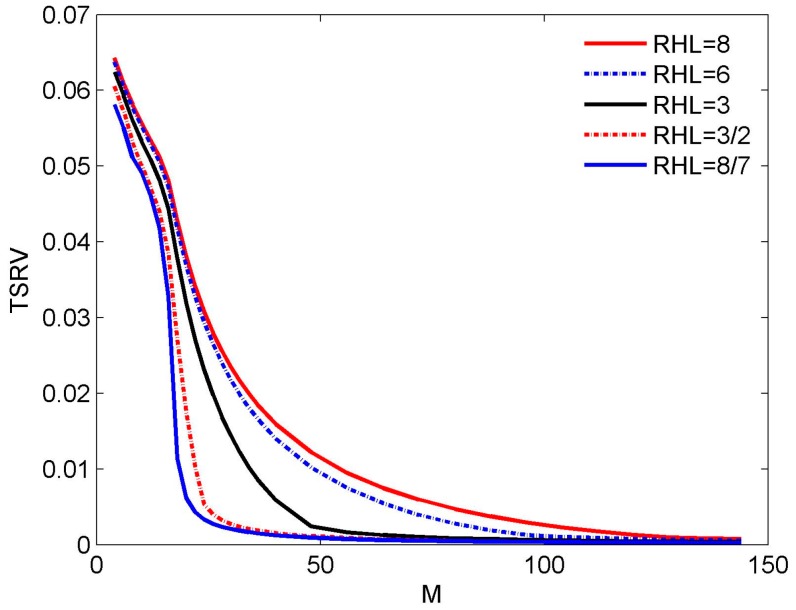
TSRV versus the number of sensors for different RHL.

To verify the result in Equation (19), we try doing more experiments under the same conditions as in Section 3.1. We go on to investigate the results for RHL = 8, 6, 3, 3/2 and 8/7, as shown in Figure 10. Combining the result in Figure 3 and Figure 10, some interesting results are obtained. For RHL = 8, 6, 4, and 3, the TSRV curve converges at about *M* = 128, 96, 64, and 48, i.e., 8*K*, 6*K*, 4*K*, and 3*K*, respectively, which accords with Equation (19). For RHL = 2, 3/2, and 8/7, the TSRV curves converge at about the range from *M* = 32 to *M* = 40, deviating from Equation (19). Nevertheless, in each of these three cases, there always exists a turning point, above which the curve degrades rather slowly. For example, for RHL = 2, 3/2, and 8/7, the turning point is at *M* = 32 (equal to 2*K*), 26 (close to 3*K*/2), and 20 (close to 8*K*/7), respectively. Therefore, Equation (19) can also be seen as a rough conclusion for the reference beam design.

The aim of the second group of experiment is to verify our analysis in Section 5.1. All the reference beams with constraints in these experiments are designed with the convex optimization toolbox CVX [21].

The first experiment is to verify the commonality of scheme proposed for reference beam design, and two examples are given here. The experiment conditions are the same as that in Figure 9 except that the reference beam type will be changed.

**Figure 11:**
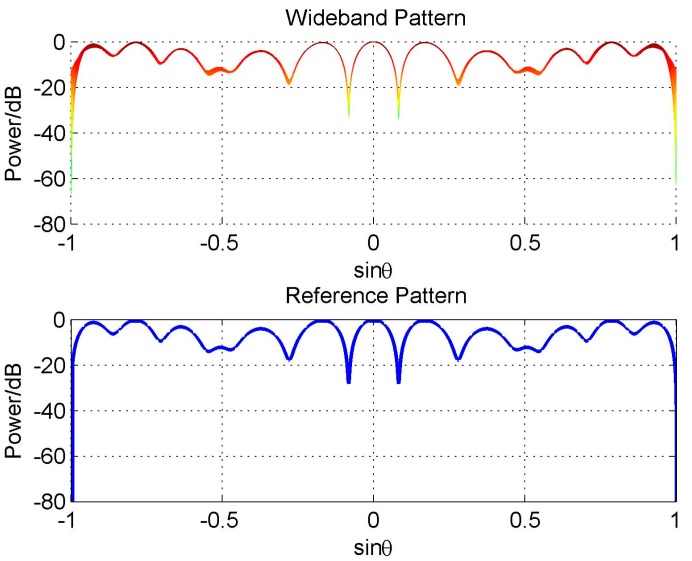
Random beam pattern design by the proposed scheme.

We firstly consider a random beam, that is, **w**_h_ is a random vector. The designed reference beam and 2-D wideband beam are shown in Figure 11. It is shown that the wideband beam has a good FI property.

**Figure 12:**
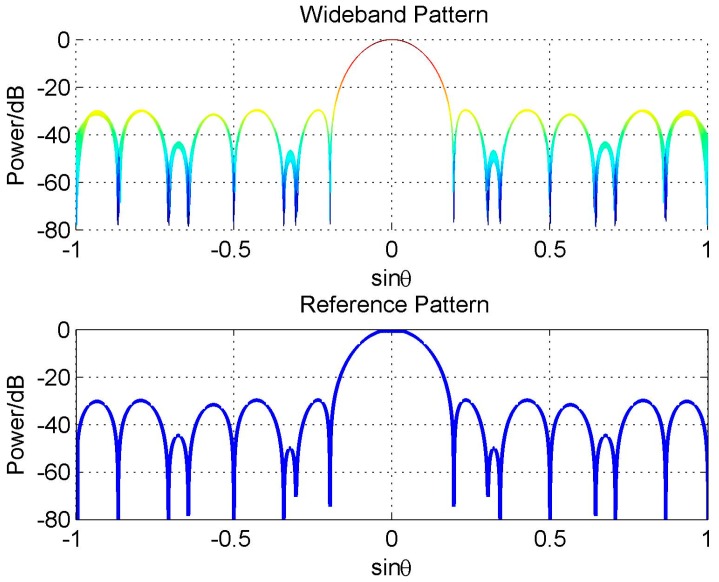
Beam pattern designed by the proposed scheme with constraints MW = 10°, SLL = −30 dB, and four nulls at 20°, 30°, 45° and −60°.

In the other example, we design a beam with MW=10°, SLL=−30dB, and four nulls at 20°, 30°, 45°, and −60°, respectively. The designed reference beam and 2-D wideband beam are shown in Figure 12. Also, the wideband beam has a good FI property.

**Figure 13:**
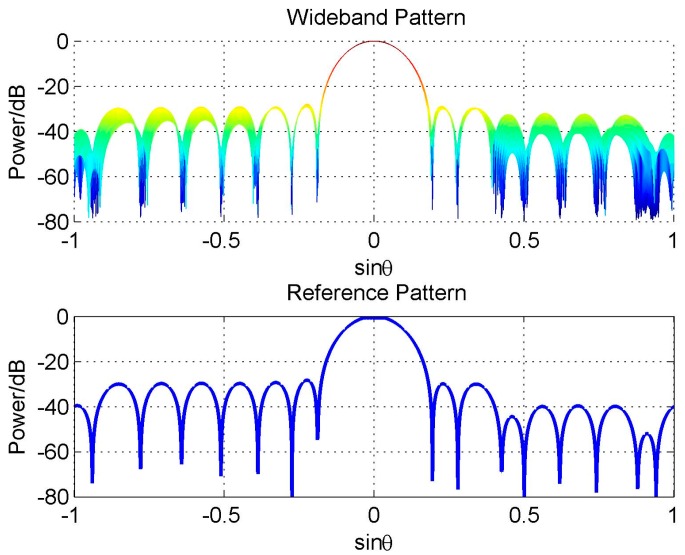
Constant beamwidth pattern design with constraints MW = 10°, SLL = −30 dB in [−90°, −12°]∪[12°, 24°], SLL ≤ −40 dB in [26°, 90°], and two nulls at 30° and 70°.

The second experiment is to verify the analysis on the reference beam design in constant beamwidth case. The experiment conditions are also the same as that in Figure 9. But in reference beam design, we set SLL ≤ −30 dB within [−90°, −12°]∪[12°, 24°], SLL ≤ −40 dB within [26°, 90°], and two nulls at 30° and 70°, respectively. The designed reference beam is obviously asymmetric, and consequently the FI property degradation occurs in the sidelobe area of the reference pattern. However, the FI property in the mainbeam is still satisfying, as shown in Figure 13.

## 7. Conclusions

The reference beam pattern design for FFT-FIB algorithm has been studied in this paper. For the simple beam, the results are shown in Equations (17)–(19). It is found that for a given beam type (window type), the reference beam pattern is determined only by the *M* and RHL. For a given reference beam, *K* is determined by the reference frequency.

For the complex beam with constraints, the designed wideband beam is always with a good FI property as long as the reference beam is symmetric. That is, if the reference beam satisfies Equation (19) and is symmetric as well, it is always suitable for FFT-FIB. To ensure the symmetry of the reference beam, the available degrees of freedom are at most *K*/2−1, but in the constant beamwidth case, the condition can be much looser.

It should be noted that this paper only concerned the broadside-mainbeam case, and did not mention the non-broadside mainbeam case. As for the off-broadside mainbeam case, the conclusions given in Section 4 are also suitable, because the analysis in this section is based on the Nyquist sampling theorem and interpolation theory which has not any special requirement on the shape of the beam. Generally, the FI property of the wideband beam designed by FFT-FIB is not that good at the frequency close to ±π. It results from the discontinuity problem [20] and has nothing to do with the reference beam design. In viewing of this, the discussion of the non-broadside mainbeam case is unnecessary and meaningless.

Now we try finding out the reason why the authors in [12] give the conclusion *M* ≥ 3*K*. We investigate the parameters for the desired signal introduced in Table I in [12]. According to the parameters (center frequency 0.29*f*_s_, and fractional bandwidth 97%), it is easy to calculate that its RHL is approximately 3. It also accords with the result in Equation (19).
